# Putting deep learning in perspective for pest management scientists

**DOI:** 10.1002/ps.5820

**Published:** 2020-04-10

**Authors:** Robert D Clark

**Affiliations:** ^1^ Simulations Plus, Inc. Lancaster CA USA

**Keywords:** artificial intelligence, convolutional neural networks, CNN, deep learning, deep neural networks, DNN, quantitative structure–activity relationship, QSAR, recurrent neural networks, RNN

## Abstract

‘Deep learning’ is causing rapid technological changes in many fields of science, and conjectures about its potential for transforming everyone's work and lives is a matter of great debate. Unfortunately, it is all too easy to apply it as a ‘black box’ tool with little consideration of its potential limitations, especially when the data it is being applied to is less than perfect. In this Perspective, I try to put deep learning into a broader mechanistic and historical context by showing how it relates to older forms of artificial intelligence; by providing a general explanation of how it operates; and by exploring some of the challenges involved in its implementation. Examples wherein it has been applied to pest management problems are provided to illustrate how the technology works and the challenges deep learning faces. At least in the near term, its biggest impact on agrochemical development seems likely to come in automating the tedious work involved in assessing agrochemical efficacy, but getting there will require major investments in building large, well‐curated data sets to work from and in providing the expertise required to assess the resulting model predictions in real‐world scenarios. Deep learning may also come to complement the machine learning methodologies already available for use in pesticide discovery and development, but it seems unlikely to supplant them. © 2020 The Authors. *Pest Management Science* published by John Wiley & Sons Ltd on behalf of Society of Chemical Industry.

## Graphical abstract

To put deep learning into a broader context, this Perspective relates it to older machine learning tools; explains the basics of how it works; and explores some of its limitations.

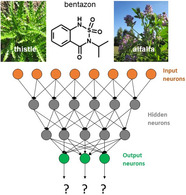

## 1 INTRODUCTION

Broadly speaking, ‘artificial intelligence’ (AI) can be characterized as any technology intended to mimic the human mind's ability to perceive, to analyze, to reason or – ultimately – to be creative. It has been a growing presence in our daily lives for many years, but until recently its effects have stayed mostly in the background. We have all benefitted for years from automated solutions to tedious pattern recognition problems such as optical character recognition for mail sorting and check processing. Automated translation has been visible, too, though mostly as a source of humorous examples that illustrate the technology's limitations. AI has loomed larger recently due to the publicity surrounding one approach in particular: ‘deep learning’ (DL) programs that consistently outperform human grandmasters at games like Go,^1^ and drive the development of autonomous vehicles.^2^, ^3^ This progress has led some to predict a new wave of technological change that will have social effects on the scale of the Industrial Revolution that began at the end of the 18th century.^4^ A few of those with extensive experience in the field, however, have pointed out that applying the tools is not as easy as it may seem; applying them correctly, at least, is not.^5^, ^6^

DL has been slow in coming to pest management *per se*, but it has begun to make itself felt in agriculture in general.^7^ Exemplary applications include insect pest identification,^8^, ^9^, ^10^, ^11^, ^12^, ^13^ identifying weeds in crops,^14^, ^15^, ^16^ and differentiating between plant diseases.^17^ Such tasks are directly analogous to tasks DL tools were developed to solve, i.e. they are not fundamentally different from distinguishing cats from dogs. More difficult and specialized applications like quantitatively assessing plant damage in glasshouse screens or agrochemical field tests will likely be forthcoming. Enhancing classical pesticide quantitative‐structure activity (QSAR) models^18^, ^19^, ^20^, ^21^, ^22^ is likely to be explored as well, if recent publications in the drug discovery area^23^ are any guide.

This article is a Perspective, not a survey of the DL literature, recent pest management applications, or a tutorial. Readers interested in an exhaustive survey of agricultural applications of DL should consult the excellent reviews of the subject published in 2018.^7^, ^24^, ^25^ This essay is instead a collection of observations from a researcher working at the periphery of DL – observations that are informed by three decades of work on machine learning (Box 1) in general, including a decade devoted to agrochemical research and development. I start by looking backward, putting DL into historical and technical context by describing how the method evolved, how it differs from classical statistical modeling approaches (Box 2), and some fundamentals of how it works. A discussion of the general challenges it faces follows. I conclude with some recommendations as to how and where DL might be productively applied in agrochemical research.

Much of the DL literature is steeped in jargon, but there are many excellent general reviews available that range from the very general^5^, ^26^, ^27^ to ones that focus on pharmaceutical^23^, ^28^ or agricultural^25^ research and development. The examples of pest management applications discussed herein^9^, ^11^, ^29^, ^30^ were chosen because they include good summaries of previous work in their respective areas.

I have tried to favor references that are relatively accessible to general audiences but still provide detailed and technical references for anyone who wishes to get into the details. That said, the field of DL as a whole is too big and is evolving too fast for any publication to be considered comprehensive or definitive. It follows that any projection of general or specific future prospects – including this one – should be taken with a grain of salt.

2

Box 1 **Some machine learning terminology**

Machine learning encompasses a variety of computational tools that model the properties of an existing data set in such a way that the properties of new examples can be accurately predicted. It is often cast as a proper subset of AI, but most reviews consider population‐based techniques like multiple regression (see Box 2) to be examples of machine learning.^7^, ^39^ The statistics community distinguishes between such statistical modeling methods and purely algorithmic ones, however.^57^

Decision trees, random forests and neural networks are described pretty thoroughly in the text (see section entitled ‘Decision trees’). For further discussion of them and other machine learning methods not alluded to here, see one of the many recent reviews available on the topic.^7^, ^39^

Classical multiple linear regression (MLR) takes an ordered set of input values (i.e. a vector of descriptors) and the corresponding endpoint values (i.e. a data matrix) and directly calculates the high‐dimensional straight line that minimizes the sum of squared endpoint deviations from that line. If the dependence of the endpoint on the descriptors is in fact linear, the distribution of noise in the endpoint values is independently and identically distributed (IID), and the training pool represents a random sample of the population of interest, then an MLR model is expected to be statistically optimal. Moreover, if the descriptors are statistically independent variables and the underlying relationship between them and the endpoint is linear, the magnitude of the coefficients obtained reflect the degree to which each ‘explains’ the endpoint.

Tools exist for dealing with cases where the underlying relationships are known to be non‐linear or where the distribution of noise across the sample is more complex, but such generalized multiple regression techniques require correspondingly more complex assumptions about the functions and distributions involved. Moreover, they still generally rely on an assumption that the descriptors are statistically independent.

Unfortunately, the descriptor sets typically encountered in practice are highly intercorrelated, sampling is rarely random in any realistic sense, and experimental errors are often neither uniform nor independent of the endpoint even to a first approximation. Moreover, if the pool of potential descriptors is larger than the number of observations in the training pool, the resulting data matrix is indeterminate – i.e. the optimization problem will have no unique solution.

Principal components analysis (PCA) entails extracting a series of linear combinations of descriptors that are not correlated with one another. The ‘latent variables’ obtained are generally better suited to MLR than are the correlated input descriptors, and using a subset of them for that purpose is termed PCR. The term ‘principal components’ refers to the fact that the latent variables are prioritized by the degree to which they capture the overall information content (variance) in the original descriptor matrix. Having more descriptors than observations is handled by keeping the number of latent variables small.

Partial linear regression (PLS; also known as projection to latent structures) is similar to PCR but takes the correlations between the descriptors and the endpoint into account when extracting and prioritizing vectors of ‘latent variables’ for use in regression analysis.

Support vector machines (SVM; originally called ‘support vector networks’^58^) are models in which a rather complex non‐linear buffer zone (the ‘support vector’) is identified rather than a linear regression line. The distribution of endpoints away from the buffer zone represented by that band have little direct influence on the optimality of classification models, which reduces the sensitivity to outliers.

The details of how they accomplish this and the sense it which it is optimal lie beyond the scope of this Perspective; please see the reviews cited above for details.

An activation function is the function applied to the aggregated inputs to a hidden neuron to generate the output to another hidden or output neuron. Sigmoidal activation functions (typically the logistic or hyperbolic tangent) are used for shallow ANNs. Rectilinear activation functions (zero below a threshold and linear above it) are more common for DNNs. The long short‐term memory functions often used in RNNs can be thought of as a kind of activation function.

Wavelet analysis is similar to PCA in that it involves decomposition of the descriptor data matrix into a series of complementary combinations of the input descriptors, but it differs in that the descriptor combinations are not constrained to be linear. It is most typically used with spectral data, e.g. to monitor stress in wheat due to aphid infestation using infrared reflectance spectroscopy.^41^

3

Box 2 **The two cultures of modeling**

Machine learning methods (see Box 1) have been characterized as falling into two different groups. The decision tree, random forest, and neural network models discussed in the body of the text focus on generating outputs that will match the endpoint values associated with sets of inputs that have yet to be encountered; where the data came from tends to be a secondary consideration. Such models were categorized as ‘algorithmic’ at the turn of the century^57^ but ‘descriptive’ seems equally or more appropriate now.

The traditional modeling culture relies on more familiar tools like analysis of variance (ANOVA), MLR, and PLS, methods that focus on elucidating the underlying relationships (associative and causal) between endpoint values (dependent variables) and potential inputs (descriptors) that are specific to each data set. Its practitioners treat each data set as a sample from the population about which the modeler wants to make statistically valid inferences. This focus presumes knowledge about how data are distributed across endpoint values and the attributes of interest (‘descriptors’), as well as how those attributes relate to each other. These are niceties with which more recently developed approaches are not particularly concerned. Such approaches were called ‘data modeling methods’ in the 1990s.^57^ but ‘statistical methods’ seems more apt today. When the assumptions made about the distributions involved are correct, one can make good predictions based on a minimum amount of information. Moreover, it is often possible to calculate how confident one can be in those predictions. Conversely, the conclusions drawn can be very wrong if incorrect assumptions are made about the underlying population. Some degree of residual deviation from the model in the form of random noise is expected and accounted for.

Simple statistical models work well for linear relationships and endpoints for which residual deviations are normally distributed, provided the data set is a random sample of the population of interest. The degree of correlation between the descriptors in the target population must also be taken into account when interpreting the statistics they produce. If it is low enough, they can be treated as independent variables, but if it is high it is often necessary to generate a reduced set of uncorrelated descriptors – i.e. ‘latent variables.’ Coming to a correct conclusion depends on getting the details of the distributions involved correct, which generally makes statistical methods less amenable to automation than are those that make use of descriptive modeling methods.

## 4 THE EVOLUTION OF NEURAL NETWORKS

### 4.1 Decision trees

**Figure 1:**
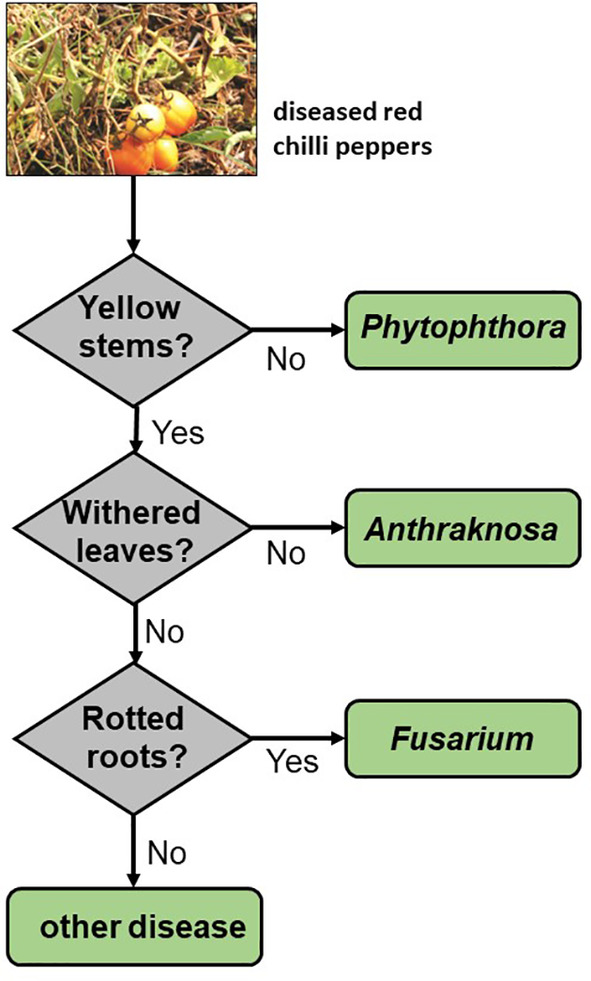
Simple classification model to distinguish among some fungal infections of red chili peppers. Adapted from the expert system described in reference 30.

Arguably the earliest AI programs consisted of a series of questions. The response to each question obtained by soliciting input from a user or by analyzing the input subject of interest (e.g. a disease^31^) could be used to determine which question or questions needed to be asked next. The questions used in such programs and their placement in the hierarchy were originally based on interviews with experts in the field of interest, hence they came to be known as ‘expert systems’. A simple example based on an expert system for diagnosing fungal disease in red chili peppers^32^ is shown in Fig. 1. Once enough information had been obtained to come to a final decision, a classification label (e.g. ‘probably *Fusarium*’ or ‘probably *Antraknosa*’) would be generated and communicated back to the user.

Unfortunately, asking experts to reduce the process by which they come to a decision to a series of questions and answers sometimes fails to capture everything that is actually involved in the process, even when that process is a relatively simple one and the experts being interviewed all see it in a similar way. At least initially, such a scheme is almost guaranteed to work poorly for the kind of complicated systems where such automation is most useful. This was addressed by running many subjects through the program, comparing the outputs generated by the program with those obtained from a panel of experts, and then tweaking the questions and their arrangement until the degree of agreement was satisfactory.

In many cases it turned out to be easier to simply ask experts to provide the important questions (e.g. ‘Is it a caterpillar?’ or ‘How many legs does it have?’ when identifying insects) and collect the answers to each question for a set of examples to use in building a model, i.e. to create a ‘training pool’. Data from the training pool were then fed into the computer as ordered sets of values (i.e. ‘input vectors’) along with the corresponding endpoint (i.e. the desired output). Internally, ‘Yes’ and ‘No’ answers were typically represented by ‘1’ and ‘0’ in the input vector. The computer program then explored a variety of arrangements of the questions (‘models’), applying them to the examples to determine which model produced outputs that best matched the endpoint values. The process included setting specific numerical decision thresholds (e.g. six *versus* eight legs) for non‐binary decision points.

The models that result are called ‘decision trees’.^28^ The variation most commonly used today is called a ‘random forest’ model. It consists of an ensemble (‘forest’) of decision trees, each trained on a random subset of the training pool data and taking molecular descriptors as inputs. The model's output is the average (or consensus, for classification problems) of the individual tree predictions. The degree of concordance of the predictions is taken as an indicator of the average prediction's uncertainty. Note that it is not a direct estimate of the uncertainty, however, because the models in an ensemble trained to a common endpoint are not likely to be statistically independent.^33^

### 4.2 Artificial neural networks

**Figure 2:**
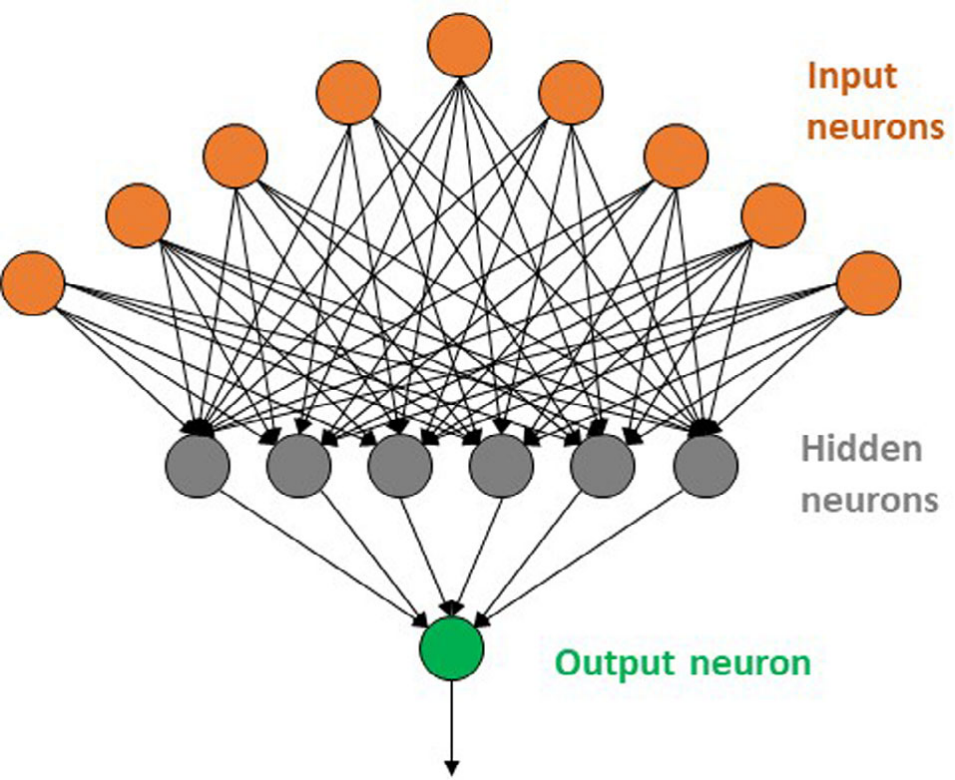
Schematic diagram of a simple fully‐connected artificial neural network with one hidden layer and a single output.

The next evolutionary step in the development of DL can be thought of as letting the computer formulate the questions themselves based on potentially relevant input vectors and their corresponding endpoint values. This can be done by setting up a network of interconnected nodes arranged in several layers (Fig. 2), with each node in the first layer corresponding to a particular input attribute and with each node in the last layer corresponds to one kind of output. Nodes in the intermediate ‘hidden’ layers are connected to nodes in the layer below them and to the nodes in the layer above them but not to others in the same layer (Fig. 2). Each incoming connection has a weight and each node has an associated offset (‘bias’). The output values (‘weights’) for the outgoing connections from a hidden node are obtained by applying a non‐linear ‘activation function’ to the summed weights of its incoming connections minus the node's bias. Each hidden node can be thought of as a question about the values of its incoming connections, and its output as an answer to that question that depends on the bias value for that node. Unless a simple step function (e.g. ‘0’ if the arithmetic combination of incoming values is less than the bias and 1 if it is greater than or equal to the bias) is used as the activation function, the ‘answer’ is a continuous number rather than the binary positive (‘Yes’) or negative (‘No’) typical of decision trees. This accommodation of fuzzy answers allows such networks to better handle subtle distinctions than decision trees that are restricted to ‘crisp’ answers at each step can.

Such systems were used, among other places, in pesticide QSAR analysis.^18^, ^22^ They were referred to as ‘multilayer perceptrons’ or as ‘artificial neural networks’ (ANNs) because their structures were inspired by the way people thought the brain processes inputs when recognizing patterns and making decisions. In that scenario, the network nodes are analogous to neurons and the connections are analogous to synapses. The analogy turned out to be seriously flawed^27^ but the nomenclature stuck, and the network nodes have been referred to as ‘neurons’ since then.* Similarly, ANNs are said to ‘learn’ the relationship between inputs and outputs as the weights and biases are iteratively adjusted until the outputs obtained are in good agreement with the corresponding endpoint values or classifications (‘labels’). Such anthropomorphic terminology can be misleading but it is standard for the field and so will be used hereafter without further apology or qualification.

It is worth noting in passing that decision trees start from a single initial question and branch downwards from there, with each terminal node in the tree (each ‘leaf’) representing an output. Classical ANNs have an inverted architecture, where inputs feed into neurons at the ‘top’ of the network (the leaves) and the branches in the network eventually converge down to a single output node.

The ANNs developed for QSAR applications in the1980s generally employed a sigmoidal activation function, and processed the training pool as a block throughout the training process. They typically had a single hidden layer of neurons and were ‘fully connected,’ meaning that each neuron is connected to every neuron in the layer immediately above it and to every neuron in the layer immediately below it. There were no connections between neurons within a layer, however, nor to neurons not in layers adjacent to its own. A schematic representation of one such network is shown in Fig. 2. There is an input neuron for each element in the input vector of descriptors and a single output neuron that yields a value for regression models or a classification label for binary endpoints. This kind of architecture has been and still is frequently used in QSAR work.^18^, ^34^

Training ANNs is a stochastic process rather than a fully deterministic one, with network weights and biases randomized before training begins. Input vectors (e.g. molecular attributes relating to size, lipophilicity, polarity, net charge, and the presence or absence of particular substructures) are fed into the network and the outputs compared to the observed endpoint values (e.g. herbicidal, insecticidal or fungicidal potency). Traditionally, discrepancies found are fed back up through the network (‘back propagated’), and the weights and biases modified so as to reduce the discrepancies. The cycle iterates until the outputs are in good agreement with the endpoints. Iterating all the way to convergence is not a good idea because the network tends to become overtrained, memorizing noise not relevant outside the training pool as well as or instead of the signal. Learning random or incidental correlations can also result. Some kind of early stopping technique needs to be employed to prevent this. A good approach is to verify at the end of each iteration that predictions for some training pool compounds not directly used in training (a ‘verification set) keep getting more accurate as the weights continue to get optimized.^35^

## 5 DEEP NEURAL NETWORKS

**Figure 3:**
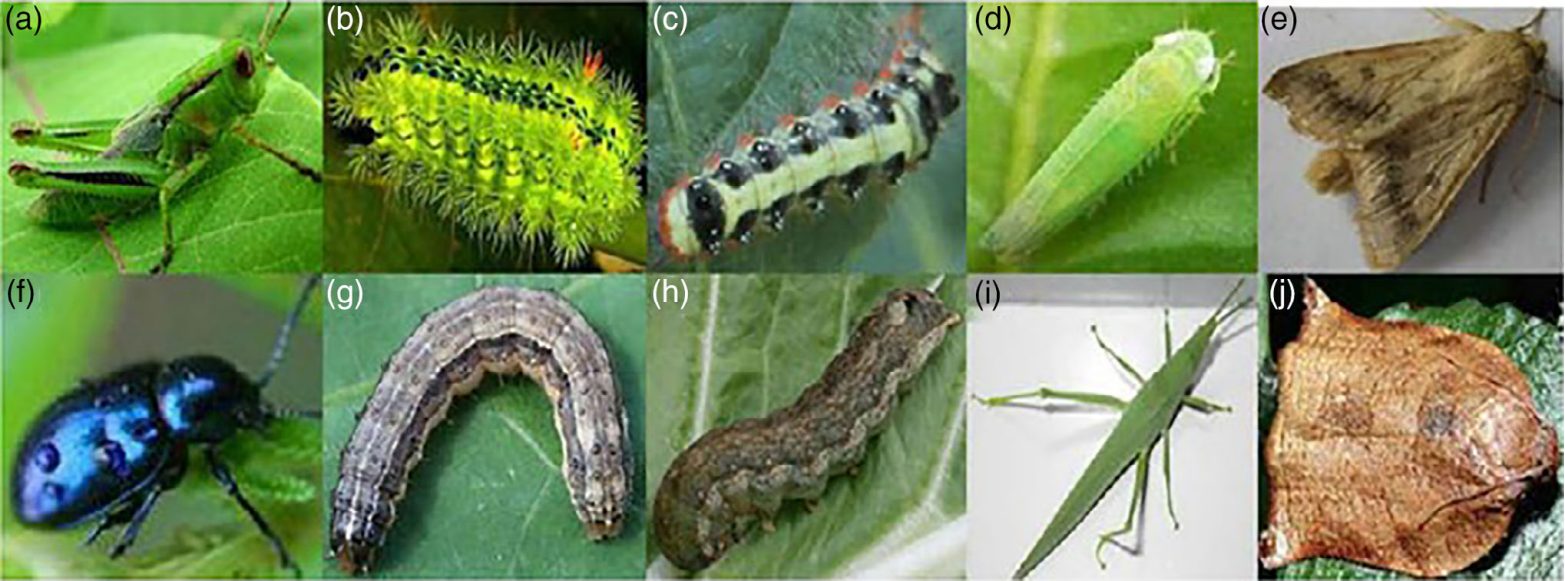
The panel of example images the ten associated labels used in a recent study on using a convolutional neural network (CNN) to classify images of insect pests associated with tea plants. Insects were identified by the authors as (a) *Locusta migratoria*, (b) *Parasa lepida*, (c) Gypsy moth larva, (d) *Empoasca flavescens*, (e) *Spodoptera exigua*, (f) *Chrysochus chinensis*, (g) *Laspeyresia pomonella* larva, (h) *Spodoptera exigua* larva, (i) *Atractomorpha sinensis* adult, and (j) *Laspeyresia pomonella* adult. [Figure reproduced with permission from Dawei *et al. J Sci Food Agric* 99: 4524–4531 (2019); reference 9.]

Classical ANNs are ‘shallow,’ in that they typically have only a single layer of hidden neurons. They work quite well when the inputs take the form of a true vector – i.e. the meaning of the tenth input value is the same for all examples in the data set. It turns out that architectures with many hidden layers of neurons (deep neural networks, or DNNs) can make sense of input streams that are only partially ordered, i.e. for which the meaning of the tenth input value for one example depends on the values of inputs for that example that are ‘close’ to it in some sense, but is not necessarily related to the meaning of the tenth input value for other examples. The color and intensity of pixels encountered in a photographic image are good examples of such partially ordered inputs: an image of the blue leaf beetle *Chrysochus chinensis*, for example,^9^ will include many blue pixels very similar in color to many of the pixels near it. The presence of such a blue patch is a strong indicator that the image in question is of blue leaf beetle in the picture, especially when the other pest options are green or some shade of brown (Fig. 3).

### 5.1 Convolutional neural networks

**Figure 4:**
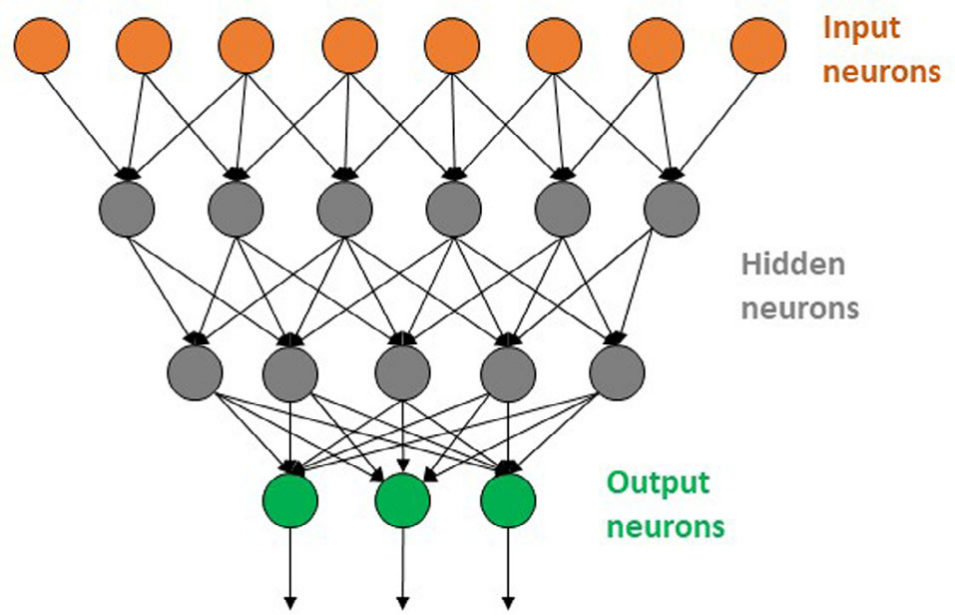
Schematic diagram of a simple deep neural network (DNN) with two hidden layers and three outputs.

Many of the DNNs responsible for high‐profile successes operate on two‐dimensional (2D) images, which convolutional neural networks (CNNs) were designed to process. They are the easiest kind of DNN to represent schematically, and an example is shown in Fig. 4. Unlike the highly structured attribute inputs typically used in QSAR analysis, the information in images is only locally structured into characteristic ‘features.’ If an insect pest has eyes, antennae, wings, legs and spots, those features are going to lie close to one another relative to unrelated features in the image, and the characteristic combination of more elemental features that make up those features – i.e. edges, arcs, and patches of color – lie even closer together. The exact location of each feature in a particular image, however, is subject to rotation, translation and scaling (zoom) effects. Hence each neuron in the first hidden layer of a CNN only receives inputs from pixels in a small part of the image, and the same partial connectivity applies as one moves down through the network, though the definition of ‘local’ changes. The result is that the upper layers in a trained network recognize basic features such as edges, lines and patches of proximal pixels similar in color. Information about the relationship between basic features percolates down to deeper hidden layers that recognize composite features like a caterpillar's legs and stripes or spots.^26^

The process of deciding which images should be included in the training pool or in the test set against which the final model is evaluated can have major effects on training outcomes. Selection may be semi‐automated but nearly always includes some manual elements, either on the part of the person compiling the data set or indirectly – often before the image is posted on the internet.^29^, ^30^, ^36^ In addition, images are preprocessed to some degree before being submitted to the CNN, either by applying automated tools or manual selection to pick out objects (weeds or insects of interest) in training pool images.^10^, ^14^, ^16^, ^30^

### 5.2 Recursive neural networks

Natural language and other sequentially organized data are processed by a different kind of neural network. Long‐range but variable relationships in such input streams – between a verb and its object when extracting data from literature textual sources, for example – can be as important as local ones if not more so. The recursive neural networks (RNNs) developed to process such data sets contain feedback loops (recursions) that allow them to remember some past inputs and to forget others as it proceeds down the input stream. They can be difficult to represent schematically, and interested readers are referred to a recent review^37^ for technical details. Most current implementations incorporate a special kind of network substructure called a long short‐term memory (LSTM) cell, which can be thought of as having a special activation function that modulates its inputs and outputs based on some inputs encountered earlier in the input stream.

RNNs may eventually be the kind of DNN that has the greatest influence on the practice of QSAR in general.^38^ That is because they can generate a vector of customized molecular descriptors directly from molecular structures by parsing their representations in an artificial language (the simplified molecular input line entry system, or SMILES) that captures their connectivity.^39^ The existence of rings or branching in molecules introduces the long‐range relationships that necessitate the use of RNNs for processing such inputs.

Some preprocessing is done to reduce the amount of learning the DNN has to do. It is generally canonicalized to some degree before ‘tokenizing’ it by replacing multi‐character elemental symbols like ‘Cl’ and ‘Br’ with special single characters, for example. A special character is then inserted at the front and another at the end of each input SMILES, and dedicated ‘null’ characters are then added to the string to pad it out to a fixed length.

### 5.3 Transfer learning

DNNs require large numbers of diverse examples if they are to perform well outside the data set used to train them,^5^, ^40^ but the amount of data available for a given endpoint of interest is often relatively limited. Such cases are often addressed by training a deep network on a large data set that is available (e.g. chemical structures from PubChem or general set of internet images^10^), with (‘supervised’) or without (‘unsupervised’) a generated target property such as hydrophobicity estimates from an existing QSAR model. In the unsupervised case, the network is training by running it ‘backwards’ to see how accurately it reproduces the input SMILES strings from the output vectors of descriptor values.

Supervised models are then retrained on the smaller data set using the endpoint of interest and keeping all parameters fixed for all but the bottom‐most layers of the network. Outputs from unsupervised models represent a condensed representation of the input data and can serve as input descriptors for simpler neural nets or other machine learning tools. These can be seen as non‐linear analogs of the latent vector used in partial least squares (PLS) and principal components regression (PCR), respectively (see Box 1). They differ in that they represent a non‐linear combination of the input descriptors; in that they are more akin to wavelets.^41^

Such descriptors are produced without direct human intervention. In that sense, they are fully ‘synthetic.’ Work done to date suggests that they are comparable in usefulness to artisanal QSAR descriptors used in the past, but it remains to be demonstrated that the models built upon them are robust. If experience shows that they are superior, they will represent a full realization of DL as a branch of AI, in they can be seen as expert systems in which all questions were formulated by a computer without benefit of direct human input. If it does not, they will join wavelets^41^ in the machine learning tool box as an interesting and sometimes useful complement to more classical descriptors.

## 6 CHALLENGES FACED BY DEEP LEARNING

DNNs are powerful tools but they are not magic. The programs used to build them are very complex and many of the details of how the DNNs they produce actually work are generally hard to pin down, making it surprisingly easy for them to consistently produce correct answers for the wrong reasons or to the wrong questions.^6^, ^40^ Even when used for the purposes for which they were originally intended, the models produced often turn out to be brittle when carefully tested, failing in surprising ways. Somewhat ironically, one of the best ways to identify flaws in a DNN is to train a second DNN to ‘break’ it. Unfortunately, most problems of agrochemical interest are somewhat different from those that DNNs were originally created to address, which makes it harder to be sure that the optimization criteria used are appropriate to the task at hand.^40^

### 6.1 Activation functions

DNNs were rarely used until recently for several reasons. First, those that employ the sigmoidal activation functions used in shallow ANNs tend to be difficult and computationally expensive to train, especially for large data sets. For one thing, advances in processor technology have increased the amount of affordable computational power enormously. For another, DNNs now typically employ rectified linear activation functions (where *f*(*x*) = 0 for *x* < 0 and *f*(*x*) = *x* for *x* ≥ 0) in place of sigmoidal activation functions. Either alternative reduces problems with the gradient calculations upon which training depends.^42^ The change also means that relationships between inputs and outputs in deep networks are piecewise linear rather than being smoothly non‐linear, but this seems unlikely to have much practical effect. More recently, hybrid ‘Swish’ activation functions in which linear and sigmoid functions are multiplied together have been employed.^43^

### 6.2 Overfitting

Secondly, fully connected ANNs having even a few hidden layers have a large number of independently adjustable parameters, usually more – and often many more – than the number of observations in the data sets available, at least for the sorts of problems encountered in pest management. Having so many adjustable parameters makes it easy to generate a model that has learned the noise – random and systematic – that is always present in real world data alongside of reliable information. The fact that CNNs are not fully connected reduces the number of adjustable parameters in the network. Conversely, training a single network against multiple target outputs or labels simultaneously effectively increases the number of observations disproportionately more than the number of adjustable parameters. The number of adjustable parameters remaining is still typically large compared to the number of observations in the training pool in both cases, however.

If the performance of a network having an excessive number of independently adjustable parameters is fully optimized, the final model is guaranteed to memorize incidental details from the training pool as well as general trends – i.e. it will become ‘overfitted.’ Overfitting is problematic because it can compromise the ability of a model to make accurate predictions for examples that are different in some way from the examples used to train it, i.e. to ‘generalize.’ When an overfitted model is applied to a test set drawn from outside the training pool, for example, it will typically perform more poorly than it did on training pool data – sometimes exceedingly so.

Statistical modeling methods (Box 2) can address overfitting by applying regularization techniques that modify the optimization criteria in a way that directly drives the model towards simpler forms. Less direct, ‘implicit’ regularization techniques are usually used^35^, ^44^ to address the overfitting challenge in DNNs. The methods used are empirical, and the measure of improved generalization is empirical as well: how well does the model perform on a representative (‘test’) subset?

One common approach is to only look at a subset of the training pool in each training iteration for the network rather than at the whole pool. Doing so presents the program with a constantly moving target during the training process. The expectation is that consistent trends will show up in all subsets and be reinforced as a result despite the constant perturbation, whereas random noise will fluctuate around zero and tend to cancel out.

‘Dropout’ is used as well. This involves setting weights for a random subset of the neurons in the network to zero as each observation in the training set is presented during training, so that each observation ‘sees’ a somewhat different model. Once the model is completely trained, a prediction is made by weighting each neuron in the final model by its assigned probability of having been dropped during the training process. This yields a prediction that approximates the value one would obtain by averaging all possible alternative models with the specified distribution of dropped neurons.^45^ The analogy to random forest models – where multiple models are built from random samples of the observations and of the descriptors – is intriguing but inexact.

Extensions of dropout include pruning and batch normalization. Pruning compresses a model by setting the weights for less important neurons to zero, a process analogous to backward elimination of variables in linear regression (Box 1). Batch normalization, in contrast, is a smoothing process in which weights are rescaled within layers in a way that reduces the sensitivity of the output to changes in individual weights.

Unfortunately, these techniques do not always avoid memorizing incidental associations in the training data (see later) even when they are effective at reducing overfitting as measured across the entire test set.^35^ Overfitting can also be missed because the test set used is not distinctive enough with respect to the training pool, which is a common problem with using random sampling to choose a test set from the very large data sets that DNNs need to be trained on if they are to be effective.^40^ A more insidious problem is that average performance on the test set risks discounting systematic errors in the input data that perturbation does not average out – i.e. biases – that get learned along with correct information.

### 6.3 Biases and incidental associations

The drive to obtain large data sets can cause problems when data from disparate sources are combined, especially if the conventions in pose, orientation and background introduce biases that lead to nominally correct predictions for the wrong reasons.^46^

Photographs found on the internet, for example, have often been pre‐selected or staged to highlight a particular aspect of the subject. The images shown in Fig. 3 nicely illustrate the potential for introducing incidental but systematic correlations between the intended target in an image and the background. Learning to differentiate between the locust *Locusta migratoria* (Fig. 3(a)), the leafhopper *Empoasca flavescens* (Fig. 3(d)), and the grasshopper *Atractomorpha sinensis* (Fig. 3(i)) is a fairly difficult task in terms of the insects themselves, but in the examples shown in Fig. 3, *A. sinensis* is pictured against a more or less uniform background. *Locusta migratoria* and *E. flavescens* are both pictured on what look to be similar leaf surfaces, though the leaf in the latter is enlarged and slightly out of focus to compensate for the insect's smaller size. Similarly, the larvae of the *Laspeyresia pomonella* (Fig. 3(g)) and beet armyworm *Spodoptera exigua* (Fig. 3(h)) are perched on leaves from distinctly different plant species.

Examination of figures from the group's previous publication in which this data set was originally described^36^ suggests that the first set of potential biases may not be fully realized in this case. In fact, *Locusta migratoria* are more often depicted on plant stems than on leaves, and *A. sinensis* is shown against a variety of backgrounds. In general, however, DNNs are quite sensitive to systematic and implicit biases in the input data that result from such incidental associations between background elements and features of the objects themselves. Early applications of DNNs in image recognition were single label classifiers. The images contained many extraneous features, but constraining the output to a single label taken from a short list of candidate labels led the networks to learn easy‐to‐recognize indirect cues rather than the more subtle but appropriate ones. A good example of this is the case where a model trained to distinguish huskies from wolves produced ‘wolf’ as the output classification for any image that contains a patch of snow.^47^

Conversely, it turns out to be rather easy to fool first‐generation image recognition DNNs because they are often not very good at handling unusual backgrounds and poses. The images used to train the programs were obtained from different poses and from many different angles around a more or less vertical axis, but that axis was oriented fairly consistently with respect to the background. When that incidental consistency was compromised – e.g. when objects are manipulated to appear skewed or tilted – the programs often fail.^48^

A recent examination of three published structure‐based virtual screening papers is a case in point. Three different benchmarking data sets had been used to evaluate performance, each composed of target proteins, known ligands and property‐matched ‘decoys’ that were presumed not to bind to the targets. The CNN programs used classified them quite well across the range of targets, but they performed almost as well when all information about which protein was the target was withheld. It turned out that the filters used to identify candidate decoys had been applied independently, but that those properties were correlated for the actual ligands in each case – and that was most of what the DNNs had learned to recognize.^49^ The rules used to select ‘actives’ and those used to select ‘decoys’ were consistent but fundamentally different, and the DNN learned the rules – i.e. the selection bias^46^ – rather than features of the protein‐ligand complexes that were characteristic of binding.

### 6.4 Problems with categorization errors

DNNs require large amounts of data if they are to train effectively, but the need for the data to also be of very high quality is difficult to satisfy on a large scale.^40^ This obviously includes making sure that individual observations are assigned to the correct category (e.g. species are correctly identified), but it also means making sure that the set of categorical labels available are appropriate to the data of interest. The latter is especially true when it entails oversimplification of the labels applied to endpoint output categories.

One kind of over‐simplification is illustrated by the decision tree shown in Fig. 1, where there is an assumption that only one of the three fungal species can be present and that the plants in question must be diseased in some way. Most DNNs, in fact, lack ‘none of the above’ labels in the training and test sets, though such situations are quite likely to be encountered in the field. Exceptions are where the number of label options is small or binary (yes or no) and the background is very simple or very consistent between examples or both. Images from insect traps with blank white backgrounds, for example, work well for automating the assessment of how severe an infestation of the brown planthopper *Nilaparvata lugens* (Stal) is.^13^ There the model picks out images from the background and determines how likely each is to be *N. lugens*. Many researchers make the assumption – often implicitly – that multiple labels are exclusive.

Over‐simplification can be addressed by training DNNs to identify multiple labels in an input image. Unfortunately, allowing individual examples to bear multiple labels has indirectly made it difficult to properly compare performance statistics for some DNNs to those for other modeling methods. Input images are usually cropped (‘downsampled’) by the program as part of the training process, and image labels get ‘refined’ as well.^50^, ^51^ An image initially labeled as containing ‘a building’ and ‘trees,’ for example, may get relabeled as also containing ‘grass’ and ‘sky’ because the program recognizes that some (sub)images are similar to images labeled as ‘grass’ or as ‘sky.’ The fact that these labels were originally omitted reflects the prejudices of the original human labelers as to which features are important. The DNN can learn that hierarchy as part of the process and use it to improve performance, but the opportunity to do so introduces biases that can distort the meaning of the statistics obtained for the test set.

Multi‐target DNNs are analogs of multi‐label image recognition networks that have been applied to QSAR problems.^38^ Most notably, multi‐target DNNs won both the Merck Molecular Activity Challenge (www.kaggle.com/c/MerckActivity) and the Tox21 competition.^52^ Classical molecular descriptors were used as inputs in both cases. Researchers at Merck & Co., Inc., analyzed the source of DNN superiority on the Challenge data and came to some striking conclusions.^53^ When a DNN was set up with multiple outputs, it did as well or better than random forest models (see the section entitled ‘Decision trees’ earlier) for each of the 15 assay endpoints (‘tasks’), similar to what had been seen in the Challenge. When the DNN was run on the 15 individual data sets – i.e. as a single task DNN – performance was comparable across the endpoints. By looking at combinations of endpoints, they found that multitask DNNs consistently had an advantage when multitask problems were addressed simultaneously for correlated endpoints – e.g. different orexin receptors. This is especially true when similar molecules are present in the training sets for the endpoints – an example of ‘transfer learning’: information from each endpoint is passed to the other, and better performance is obtained for each. Combining similar training sets and uncorrelated endpoints, however, tends to confuse the DNN and reduce performance.

A second conclusion of the article also bears noting: the Merck Activity Challenge models were evaluated against a set composed of the last compounds tested in each assay. The idea is that such chronological tests sets are representative of the future compounds that one would like to predict. The assays had been run at different times, however, so compounds in the training set for some assays were present in the test set for other, related assays; the cross‐talk from such overlaps also improved the performance of the multitask DNN. In fact, the difficulty of constructing suitable test sets for DNNs is now being recognized as a major challenge for big‐data AI in general and for DL in particular.^40^, ^49^

### 6.5 ‘Ground truth’ that is not true

The ‘ground truth’ with which DNNs are trained and against which their performance is measured is based on categorical labels or values provided by human beings, and the models obtained cannot really be more accurate than the data used to train them. They can appear to be more accurate, however, when that data includes systematic errors and the modeling technique is powerful enough to take them into account. The paper from which Fig. 3 was taken is a case in point. The image labeled as depicting a *Laspeyresia pomonella* larva (Fig. 3(g)) is identical to the one shown in the paper's final figure, where it is categorized as being an example of a *S. exigua* larva, the species shown in Fig. 3(h). This may be a typographical or data entry error or it may be a case where the image was accidentally duplicated but assigned to different categories in different places on the internet.

Regardless, the larva shown probably belongs to neither species. The image can be found† at https://www.epicgardening.com/army-worms/, where the larva is identified as being that of the fall armyworm *S. frugiperda*. A larva of the beet armyworm is shown below it and is labeled as such; that may have been why the image was miscategorized.

In this study the authors had six experts assign one of their ten labels to the insect pest images in their test sets and reported the experts' overall performance statistics as well as how well their classifications matched the assigned data set categories. Overall accuracy with respect to the assigned categories ranged from 82 to 96%, whereas they achieved an accuracy of 94% by transfer learning. All six experts seemed to confuse *Laspeyresia pomonella* with *S. exigua* larvae (depicted in Fig. 3(g, h), respectively) and four of the six seemed to misclassify the corresponding adult forms (Fig. 3(j, e), respectively). Two experts misclassified locusts (*Locusta migratoria*) as grasshoppers (*A. sinensis*) in some cases, as did the CNN.

The DL model was nominally more accurate overall than four of the six experts because it had much less trouble distinguishing lepidopterans from each other, but did mistake beet armyworm adults for blue leaf beetles in some cases. It seems likely, however, that this reflects at least in part a matter of the neural network doing a better job of mimicking biases due to systematic internet miscategorizations by non‐experts or in the course of compiling the data set.

DNNs are powerful modeling tools, and distinguishing increased ability to capture signal from learning biases and systematic errors is a moving target. Many of the problems identified to date have involved selection biases that were not identified until well after the fact and usually not by the original researchers. Some major innovations are only a few years old and have yet to be widely applied to pest management problems, so it is hard to have a handle on the kinds of biases to watch out for in our particular area.

The size of the data sets used to train deep neural nets precludes the sort of manual curation^54^ needed to avoid the problem, but it should be possible to thoroughly curate a representative test set big enough to provide something close to ‘ground truth.’ Comparing DNN performance on the ‘raw’ test set and the curated one will at least make it possible to separate spurious performance ‘improvements’ due to fitting systematic errors from those due to genuine increases in generalizability.

## 7 CONCLUSIONS

The degree to which DNNs are able to compete effectively with human judgement is likely to increase substantially in the near future, but cases where they leverage or complement human skills rather than replacing them are likely to be more important in the long run. Indeed, the experience in oncology screening is that combinations of machine and human intelligence do better than either alone, because each makes different kinds of mistakes.^55^ This is not surprising, because mimicking human decision making is not the same as reproducing it. Indeed, we probably should not want it to be, because complementary systems provide more opportunity for synergy.^56^ That will be as true in pest management science as in any other human endeavor. Certainly a tool that can be used to reduce the tedium of scoring safety in the laboratory or weed control in the glasshouse or insect damage in the field will be welcome, and the increase in raw data on pesticide activity and specificity that would result is likely to benefit more classical modeling efforts (see Box 1). That cannot happen, however, until a great deal of data has been accumulated from expert human scoring and careful curation to remove systematic errors and biases. Even then, the main benefit will probably come via transfer learning between pest species and across assay formats: the number and diversity of pest control compounds tested is likely to remain the limiting factor when it comes to applying DL approaches to QSAR analysis.

## Endnotes

*
There has been a recent shift towards referring to neurons as ‘units’ or ‘modules’, perhaps in part to reflect their increasing ‘internal’ complexity and in part to move away from (mis)identification with biological neural networks. I will stick to the ‘neuron’ terminology here for the sake of consistency.

†
Accessed 28 November 2019.
